# Glutamate 83 and arginine 85 of helix H3 bend are key residues for FtsZ polymerization, GTPase activity and cellular viability of *Escherichia coli*: lateral mutations affect FtsZ polymerization and *E. coli* viability

**DOI:** 10.1186/1471-2180-13-26

**Published:** 2013-02-05

**Authors:** Jae Yen Shin, Waldemar Vollmer, Rosalba Lagos, Octavio Monasterio

**Affiliations:** 1 Department of Physics, University of California, Berkeley; 2 Institute for Cell and Molecular Biosciences, Centre for Bacterial Cell Biology, Newcastle University, Richardson Road, NE2 4AX, Newcastle upon Tyne, UK; 3 Departamento de Biología, Facultad de Ciencias, Casilla 653, Santiago, Chile

**Keywords:** Bacterial division, FtsZ polymerization, Longitudinal/lateral interactions, ZipA

## Abstract

**Background:**

FtsZ is an essential cell division protein, which localizes at the middle of the bacterial cell to mediate cytokinesis. *In vitro*, FtsZ polymerizes and induces GTPase activity through longitudinal interactions to form the protofilaments, whilst lateral interactions result within formation of bundles. The interactions that participate in the protofilaments are similar to its eukaryotic homologue tubulin and are well characterized; however, lateral interactions between the inter protofilaments are less defined. FtsZ forms double protofilaments *in vitro*, though the key elements on the interface of the inter-protofilaments remain unclear as well as the structures involved in the lateral interactions *in vivo* and *in vitro.* In this study, we demonstrate that the highly conserved negative charge of glutamate 83 and the positive charge of arginine 85 located in the helix H3 bend of FtsZ are required for *in vitro* FtsZ lateral and longitudinal interactions, respectively and for *in vivo* cell division.

**Results:**

The effect of mutation on the widely conserved glutamate-83 and arginine-85 residues located in the helix H3 (present in most of the tubulin family) was evaluated by *in vitro* and *in situ* experiments. The morphology of the cells expressing *Escherichia coli* FtsZ (E83Q) mutant at 42°C formed filamented cells while those expressing FtsZ(R85Q) formed shorter filamented cells. *In situ* immunofluorescence experiments showed that the FtsZ(E83Q) mutant formed rings within the filamented cells whereas those formed by the FtsZ(R85Q) mutant were less defined. The expression of the mutant proteins diminished cell viability as follows: wild type > E83Q > R85Q. *In vitro,* both, R85Q and E83Q reduced the rate of FtsZ polymerization (WT > E83Q >> R85Q) and GTPase activity (WT > E83Q >> R85Q). R85Q protein polymerized into shorter filaments compared to WT and E83Q, with a GTPase lag period that was inversely proportional to the protein concentration. In the presence of ZipA, R85Q GTPase activity increased two fold, but no bundles were formed suggesting that lateral interactions were affected.

**Conclusions:**

We found that glutamate 83 and arginine 85 located in the bend of helix H3 at the lateral face are required for the protofilament lateral interaction and also affects the inter-protofilament lateral interactions that ultimately play a role in the functional localization of the FtsZ ring at the cell division site.

## Background

Bacterial cell division occurs in the middle of the dividing cell mediated by protein complex division machinery called divisome*.* FtsZ (Fts, Filamentous temperature sensitive) is the principal protein of the divisome in *Escherichia coli*, and assembles into the Z ring at the cell division site; it is bound to the inner membrane and is stabilized by FtsA and ZipA [1,2]. This event is necessary for the sequential addition of at least twelve other membrane proteins responsible for peptidoglycan assembly and membrane constriction [3]. Inactivation of the corresponding genes blocks the cell division and give rise to long multinucleated cells that fail to propagate and eventually die [4].

The Z-ring and its dynamics during the cell process have been observed by fluorescence microscopy in living cells [5] and in liposomes [6]. Although, the organization of FtsZ in the Z-ring is not known, based on the *in vitro* studies it is believed that the basic units to form the ring are the protofilaments. In order to explain the Z-ring dynamics at the molecular level, two theoretical models have been proposed. One model involves the hydrolysis of GTP to generate the force for invagination of the plasma membrane [7] and the other model proposes a microphase transition of the FtsZ filaments that is energized by lateral interactions between filaments during the constriction of the plasma membrane [8]. In both models, the lateral interactions between the protofilaments are crucial for the membrane constriction. However, the role of lateral interactions has been questioned because the interaction entropy was not considered in the formation of the filaments [9]. Therefore, the question regarding the role of lateral interactions still remains unclear.

Mutations on the lateral interface of FtsZ induce the formation of an aberrant Z-ring in *E. coli* cells and cell division can be compromised [10,11]. Apparently, the lateral interactions to form the Z-ring are also important in *B. subtilis*[12]. Recently, the Z-ring was observed *in vivo* at a higher resolution. The authors proposed that FtsZ polymerizes into short filaments that assemble into loose bundles mediated by longitudinal and lateral interactions to finally locate as a Z-ring at the division site [13]. These observations suggest that lateral interactions between FtsZ protofilaments could be important for *in vivo* formation of the functional Z ring. However, the elements that play a key role in the Z-ring formation remain unknown.

*In vitro,* lateral interactions of FtsZ stabilize sheets and rings [14] and ZipA, mentioned earlier as a membrane anchor protein, stimulates the formation of FtsZ sheets [15]. Moreover, FtsZ also polymerizes into sheets and tubules in the presence of cations such as calcium or DEAE-dextran [15-18], or by the FtsZ-interacting protein SepF [19,20]. The sheets and tubules are stabilized by lateral interactions similar to those found in tubulin polymers [14,21]. Moreover, arc-like filaments that could interact laterally have been observed by electron cryotomography at the constriction site [22], but the importance of these lateral interactions has remained controversial [23]. Ca^2+^-induced FtsZ sheets are composed of protofilaments that are aligned in parallel and antiparallel way, while in Zn^2+^-induced tubulin sheets are arranged only in an antiparallel manner. Thus, from a low-resolution sheet structure of *Methanococcus jannaschii* FtsZ, it has been proposed that two protofilaments interact through the same lateral face, in a parallel form, to produce double-stranded filaments called thick filaments, which subsequently form sheets through antiparallel interactions [15]. *E. coli* FtsZ also polymerases into thick filaments [24], indicating that these filaments can be the basic units to form sheets.

Phylogenetic and structural studies show that FtsZ is the most-conserved cell division protein and it shares structural and functional features with eukaryotic tubulins [25,26]. The heterodimer αβ-tubulin polymerizes into microtubules and two types of interactions are involved between the heterodimer subunits: the longitudinal interactions between heterodimers to form the protofilaments, and the lateral interactions between 13 or 14 parallel protofilaments to organize into the microtubule. The lateral interactions result from the contacts between α- and β-tubulin subunits of neighboring protofilaments and are mediated by central structural elements, such as the interaction of the M loop of one subunit with the H3 helix of the neighboring subunit [27]. A newly discovered bacterial tubulin, BtubA/B, also contains the H3 helix and M loop, however it is believed that the last one is a reminiscent structure from eukaryotic tubulin [28]. Interestingly, the H3 helix is highly conserved among FtsZs while the M loop is absent. Therefore, we believe that H3 helix of FtsZ can participate in the lateral interactions in a similar manner to its related tubulins.

The aim of this work was to investigate the role of the H3 helix in lateral interactions of FtsZ from *E. coli*. We mutated the highly conserved glutamate 83 and arginine 85 residues to glutamine by site-directed mutagenesis and evaluated the effect of these mutations on the viability of the cells, the cell morphology and localization of FtsZ, as well as the GTPase activity and the ability to polymerize *in vitro*, in the presence and in the absence of calcium and ZipA.

## Results

### Design of FtsZ(R85Q) and FtsZ(E83Q) mutants

**Figure 1:**
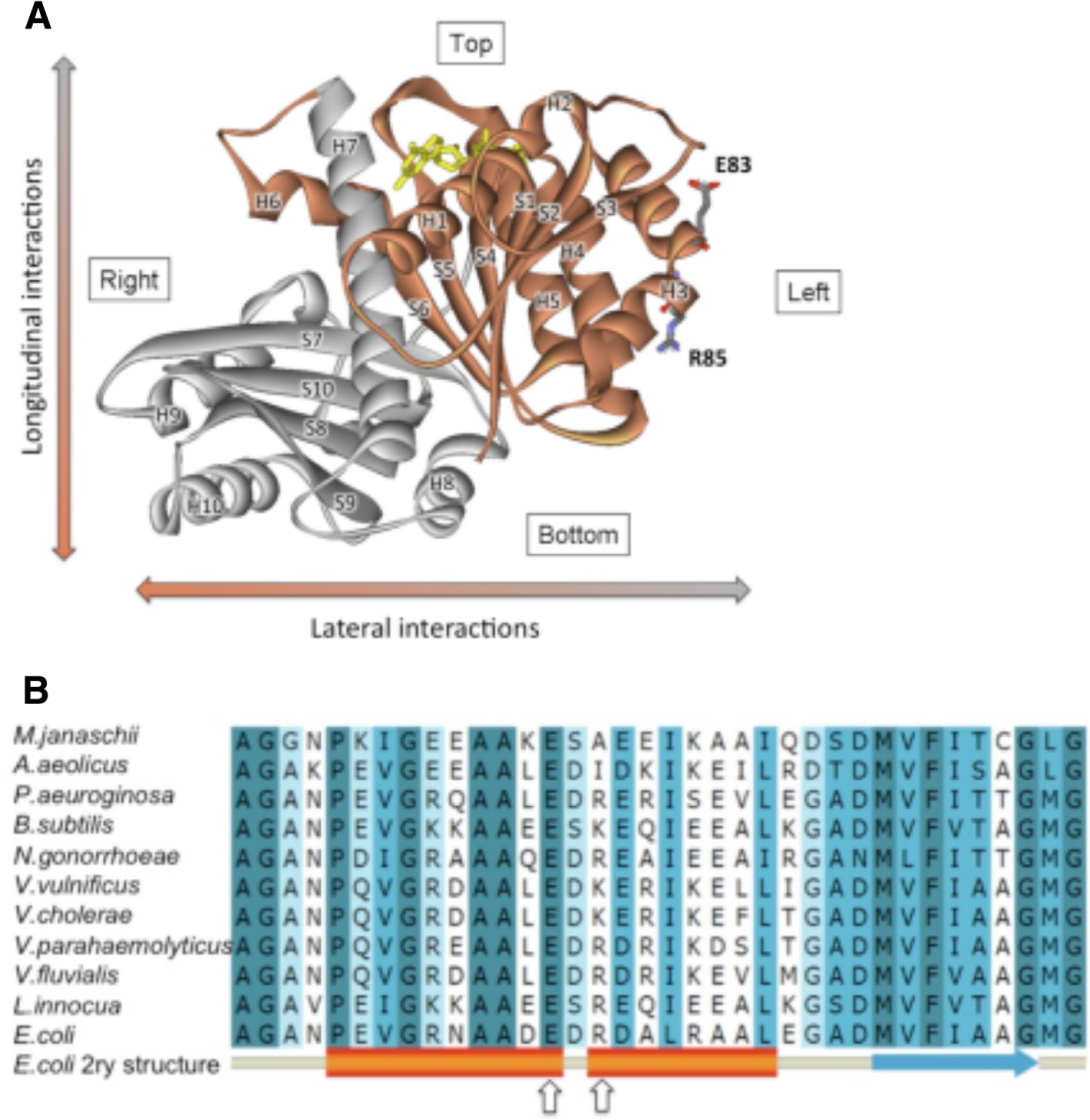
**(A) Front view of the *E. coli *FtsZ structural model.** N-and C-terminal domains are represented in brown and gray, respectively. Arrows indicate longitudinal and lateral polymerization axes and the face of FtsZ is described as top, bottom, right and left according to the orientation of GTP (yellow). Residues E83 and R85 locate in the bend within the H3 helix. (**B**) Primary sequence alignment of the H3 region of FtsZ. The most conserved positions are colored in dark blue. The secondary structure of FtsZ is shown in the last line: alpha helix, orange rectangle; beta strand, blue arrow; coil, grey. The arrows indicate residues E83 and R85 of the *E. coli* FtsZ sequence.

We choose as candidates for mutation two conserved residues located in the lateral face of FtsZ, specifically in the bend of H3 helix. This was based on the similarities in the crystal structures between FtsZ and tubulin and the degree of conservation of amino acids among FtsZ sequences. The three dimensional structure of an *E. coli* FtsZ model^a^ (Figure 1A) was compared with the structure of the αβ-tubulin heterodimer. The helix H3 of tubulin, located on the lateral face and known for the role in the lateral interactions, is also present in FtsZ. The helix H3 of FtsZ is located in the left lateral face (left, right, top and bottom face are the nomenclature defined by analogy between FtsZ protofilaments and tubulin microtubules) [27]. Helix H3 is present in most of the FtsZ sequences from gram positive and negative bacteria, mitochondria and chloroplasts. Within the H3 helix, glutamate at position 83 is 100% conserved and the positive charge of arginine at 85 is highly conserved (80% using non-redundant sequences) among FtsZ sequences from 11 different organisms (Figure 1B). Interestingly, the two residues are located in the bend of the helix H3 and are exposed to the surface of the left face. Hence, we hypothesized that these residues, E83 and R85, participate in the interactions of FtsZ to form filaments, tubules or sheets. To this end, the bend in H3 could be necessary to maintain both the topography of the monomer for the interactions and the GTPase activity in the polymer. In microtubules, the lateral interactions are principally mediated between the helix H3 from a protofilament and the M loop of the adjacent protofilament [29]. The M loop is even present in bacterial tubulin A/B, though smaller than its counterpart tubulin, and is also believed to participate in the lateral interactions to form a bacterial microtubules (bMTs) composed by five protofilaments [30]. However, FtsZ lacks the M loop hence, the lateral interactions are expected to be weaker between FtsZ protofilaments [13], hence an effect on longitudinal interactions should affect both the length and the GTPase activity of the polymers.

### Viability of *E. coli* VIP2(DE3) cells in the presence of FtsZ(E83Q) and FtsZ(R85Q)

To evaluate the functionality of the FtsZ mutants *in vivo,* the E83Q, and R85Q proteins were expressed independently and the cell viability was determined in a strain containing *ftsZ* thermonull mutant, VIP2(DE3)/pLAR9. The parental strain, VIP2/pLAR9 has been successfully employed to demonstrate that FtsZ is essential for cell division and survival in *E. coli*[31]. For the purpose of our study we modified the parental strain to create a DE3 lysogen of VIP2, that contains a copy of T7 RNA polymerase gene under the control of P*lacUV5*, so the expression of a target protein is inducible by IPTG addition. Thus, if VIP2(DE3)/pLAR9 is transformed with a second plasmid that contains the FtsZ mutants (pMFV-derivatives: pMFV-wt, pMFV-rq and pMFV-eq, see Methods), the transformed cells will contain a *ftsZ* mutant allele from pMFV-derived plasmid under the control of a T7 promoter, a wild-type allele encoded on plasmid pLAR9 (a temperature sensitive replication plasmid) and a genomic null allele. In summary, at the permissive temperature (30°C), VIP2(DE3)/pLAR9/pMFV- will express both *ftsZ* alleles (from pLAR9 and pMFV-derivatives), whereas at the restrictive temperature (42°C) pLAR9 cannot replicate, so the *ftsZ* expression originates from the pMFV-derivatives. The production of FtsZ ceases completely after 2–3 h of shifting the temperature, once pLAR9 is segregated by lack of replication. In this regard, this system is different from those utilizing a FtsZ mutant nonfunctional at 42°C, because in this case after shifting to the non-permissive temperature FtsZ will be nonfunctional almost instantly.

To simplify the nomenclature we will refer henceforth to the strain VIP2(DE3), containing pLAR9, as VIP5.

**Figure 2:**
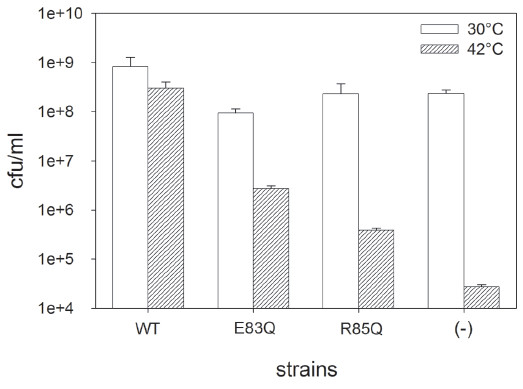
**Viability of *E. coli *VIP2 cells expressing FtsZ mutants.** Exponentially-grown cells of *E. coli* VIP5 without (−) or with plasmids that express wild-type FtsZ (WT), FtsZ(E83Q) or FtsZ(R85Q) were plated by duplicate on two LB agar plates and incubated at 42°C or 30°C. Cell viability was determined the following day. The average and the standard deviation (SD) of 3 independent experiments were obtained using the program EXCEL. The H3 bend mutations cause significant decrease in cell viability.

VIP5/pMFV-wt cell grown at 42°C (positive control) and at 30°C show similar cfu/ml in the absence of IPTG, indicating that the basal non-induced expression level from pMFV-wt (a high copy number plasmid) was enough to complement the lack of FtsZ from pLAR9 at 42°C (Figure 2). Hence, in order to avoid an excessive expression of FtsZ, IPTG was not added in all our experiments. As expected, the survival of VIP5 at 42°C (negative control) decreases about four orders of magnitude. The small survival fraction observed in VIP5 is explained as due to the reversion frequency of the chromosomal mutation [31].

Figure 2 shows that the viability of cells expressing EcFtsZ mutants E83Q and R85Q at the restrictive temperature diminished about 1.5 and 3 orders of magnitude, respectively, compared to the non-restrictive temperature. These values indicate that the cells could not support normal cell division but at the same time the EcFtsZ mutants did not completely lose functionality *in vivo*. Hence, the survival assay was useful to characterize the functionality of EcFtsZ mutants *in vivo* and the quantification of the viability of the cells expressing EcFtsZ mutants was used as an indication of the efficiency in the cell division. Consequently, the efficiency of the different forms of EcFtsZ in terms of cell viability can be interpreted as: wild type > E83Q > R85Q.

### Cell morphology and *in situ* localization of FtsZ mutants using immunofluorescence microscopy

**Figure 3:**
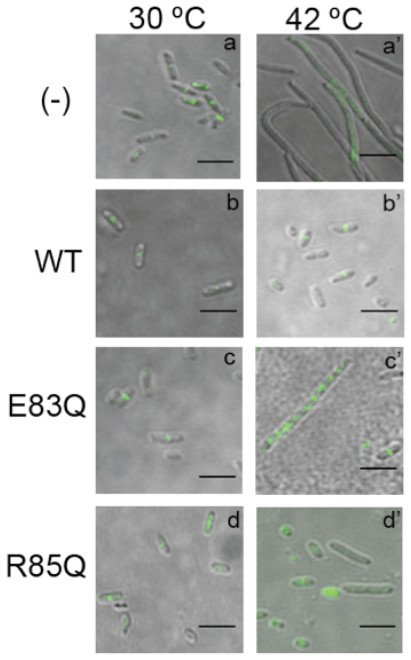
**Cell morphology and localization of FtsZ wild type and mutants E83Q and R85Q in cells with (30°C) and without (42°C) co-expression of FtsZ(WT) from pLAR9.** Immunofluorescence of FtsZ (green) overlaid with the bright field image (gray) shows the localization of FtsZ in VIP5 cells (for strain information see Methods). Scale Bar, 5μm. Immunofluorescence was performed as described in Methods. Briefly, cells were grown until OD600 reached 0.5, cultures were split in half and incubated at 30°C and 42°C respectively for 4 h. Cells were harvested, fixed and immunofluorescent labeled with anti-FtsZ as first antibody and the second antibody conjugated with Alexa 488.

The FtsZ ring is formed in the middle of the cell and is attached to the cytoplasmic membrane through ZipA [2]. In order to evaluate the effect of these mutations on cell morphology and FtsZ localization, VIP5 cells in exponential phase of growth carrying plasmids with FtsZ(WT), FtsZ(E83Q), and FtsZ(R85Q) mutants were incubated in the absence of IPTG at 30°C (control) and at 42°C. Figure 3 shows that, in the negative control at 42°C (pLAR9 is lost at this temperature) the cell division is blocked, and FtsZ localization is dispersed along the very long filamentous cells (a’), while at 30°C the cells are normal. Cells carrying the plasmid FtsZ(WT) at 42°C (b’) reverts the cell filamentation, and at 30°C (b) as expected the morphology and FtsZ localization is normal. The co-expression at 30°C of FtsZ(WT) from pLAR9 with the mutants FtsZ(E83Q) (c) and FtsZ(R85Q) (d) also display a normal phenotype. However, the expression of the FtsZ mutants at 42°C (in the absence of FtsZ(WT)) have an effect on cell division, producing filamentous cells (c’ and d’). Interestingly, FtsZ(E83Q) still localizes as discrete bands along the filamentous cells, probably at the potential cell division sites (c’). In contrast, the expression of FtsZ(R85Q) produced shorter filamentous cells with a disperse localization of the protein along the filaments, and no clear bands were observed (d’). The same experiments were performed with IPTG 0.2 mM and similar results were obtained (not shown). In order to keep the cells in exponential phase of growth for a longer time, the morphology experiments were repeated starting the culture for the temperature shift at an OD600 of 0.06. After 3 h of incubation no difference in the cell morphology of the mutants respect to the experiments presented in Figure 3 was observed.

Localization of the FtsZ mutants in the single cell population (not filaments) that appear at early stationary phase (Additional file 1: Figure S1) shows that FtsZ(E83Q) is located with greater intensity at the middle of the cell but presents some degree of dispersion, while FtsZ(R85Q) is rather distributed in a gradient shape from the mid-cell toward the cell poles. The ZipA distribution was similar to that observed for FtsZ mutants (Additional file 1: Figure S2), which suggests that the mutations in FtsZ do not affect its interaction with ZipA. Binding experiments between FtsZ (WT and mutants) and ZipA inserted into proteoliposomes confirmed this hypothesis (not shown).

In summary, the residues glutamate 83 and arginine 85 located in bend of helix H3 on the left face of FtsZ plays a role in the formation of the Z-ring (Figure 3) and consequently ZipA presents an altered localization in cells expressing the FtsZ mutants (Additional file 1: Figure S2).

### Structural characterization of R85Q and E83Q

In order to evaluate the impact of the point mutations E83Q and R85Q on the protein structure, we performed circular dichroism on purified FtsZ wt, R85Q and E83Q. All three purified proteins showed the expected molecular mass and had a very similar secondary structure content (not shown); therefore, presumably the mutations did not cause significant changes in the tertiary structure of FtsZ.

### GTPase activity of E83Q and R85Q

**Figure 4:**
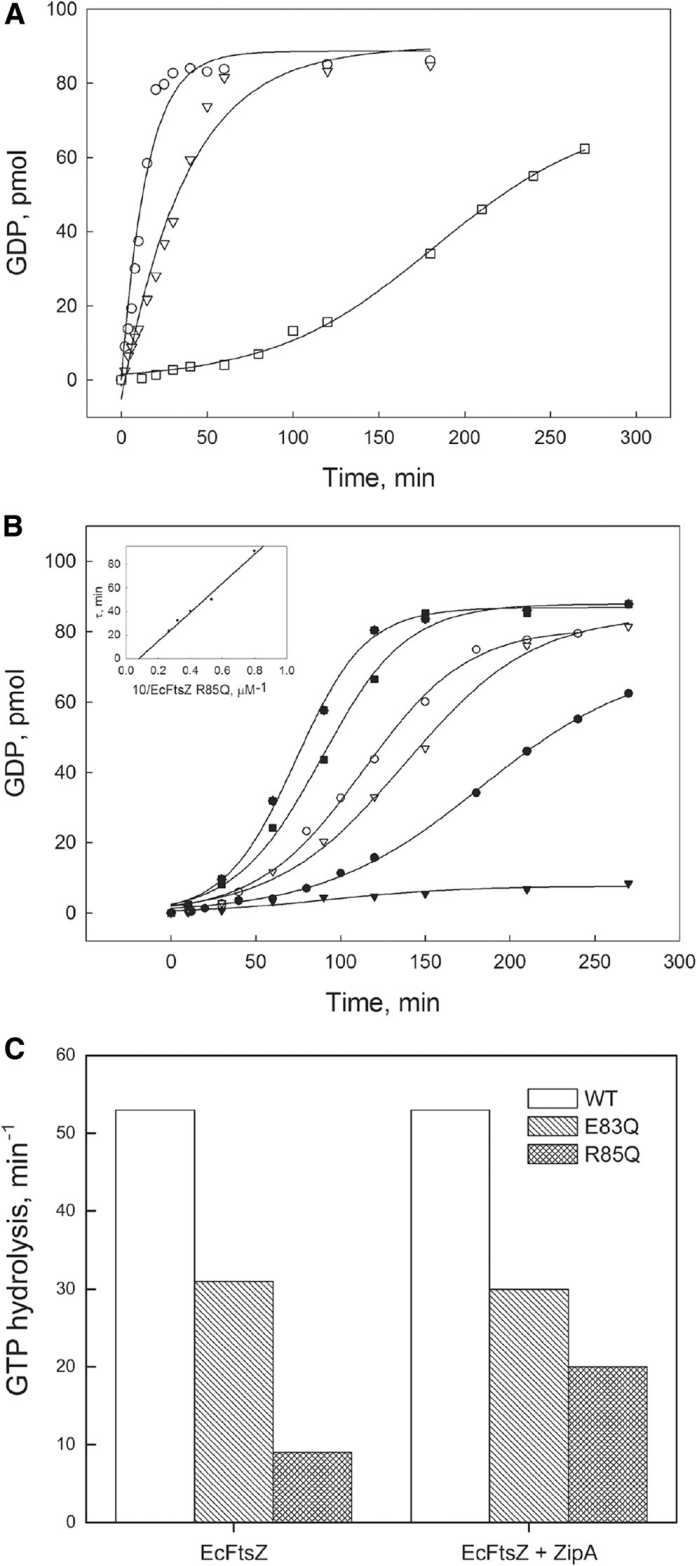
**GTPase activity of the FtsZ mutants in the presence and absence of ZipA.** (**A)** Progress curves for the GTPase activity in a solution containing 50 mM Mes pH 6.5, 50 mM KCl, 10 mM MgCl_2_ with 12.5 μM of wild type FtsZ (O), E83Q (∇ ) or R85Q (□) at 30°C. The reaction was started by the addition of GTP at 1 mM final concentration, and GDP was determined by HPLC, as described in Material and Methods. (**B**) Progress curves for the GTPase activity of R85Q. The hydrolysis of GTP was measured with the following R85Q concentrations: (▼ ) 6.2; (● ) 12.4; (∇ ) 18.6; (O) 24.8; (∎) 31.0 and 49.6 μM (). The lag time (τ) was determined as the abscissa value where the line of the steepest slope of the progress curves intersects the x-axis. The inset shows the dependence of τ on the inverse of protein concentration. The values of picomoles indicated in the ordinate axe correspond to the amount of GDP in 100 μL of the reaction mixture (as indicated in Methods). (**C**) GTPase activity of 12.5 μM wild type FtsZ, E83Q and R85Q in the presence of 12 μM ZipA; the control with ZipA alone showed no GTPase activity (not shown). Inorganic phosphate was quantified using malachite green as described in Methods and the values of the ordinate axe are multiplied by 20.

In order to understand the possible effects of the FtsZ mutations on the formation of the Z ring, we purified the mutant proteins and measured their abilities to hydrolyze GTP. The wild-type FtsZ and E83Q exhibited a constant initial velocity of GTP hydrolysis for at least 10 minutes at a protein concentration of 12.5 μM (Figure 4A). By contrast, R85Q showed a lag time (τ) of 86.3 minutes and a reduced GTPase activity. We next compared the GTP hydrolysis efficiency by calculating the apparent catalytic constants (*i.e.*, the ratio between the initial velocity and the protein concentration). Wild-type FtsZ, E83Q and R85Q hydrolyzed GTP with apparent catalytic constants of 2.90 ± 0.16 min^-1^, 1.13 ± 0.06 min^-1^ and 0.13 ± 0.02 min^-1^ respectively. R85Q was further characterized by measuring the dependence of GTP hydrolysis on protein concentration (Figure 4B). The progress curves show a sigmoidal behaviour, *i.e.*, the lag time was dependent on the protein concentration (inset Figure 4B). This lag time should disappear at the extrapolated concentration of 130 μM. The similarity of the progress curves at 36.8 and 49.0 μM of protein is probably due to GTP becoming limiting. These results suggest that the R85Q mutation does not directly alter the rate of GTP hydrolysis, but rather affects FtsZ polymerization, which is known to induce GTPase activity. Therefore, the effect is reversed when the concentration of R85Q is increased to stimulate polymerization.

The GTPase activity of FtsZ wt and E83Q is not affected by ZipA, which is in agreement with previous study [2]. Interestingly, ZipA increased around twofold the GTPase activity of R85Q (Figure 4C and Additional file 1: Figure S3b), suggesting that ZipA stimulates the longitudinal interactions of R85Q protofilaments during polymerization, similar to the effect of MAPs on microtubules [32].

### Polymerization of FtsZ mutants with and without ZipA

*In vitro,* FtsZ polymerizes into protofilaments in the presence of GTP [33], and these protofilaments associate to form bundles in the presence of ZipA [15]. Single protofilaments are monomers that interact longitudinally whereas double protofilaments (thick filaments) consist of two parallel protofilaments interacting laterally [24]. Antiparallel lateral interactions of these double filaments induce the formation of sheets or bundles [34]. In order to evaluate the effect of the mutations on the polymerization capability, we followed the polymerization of FtsZ mutants by 90° light scattering at 350 nm and the polymers morphology by electron microscopy.

**Figure 5:**
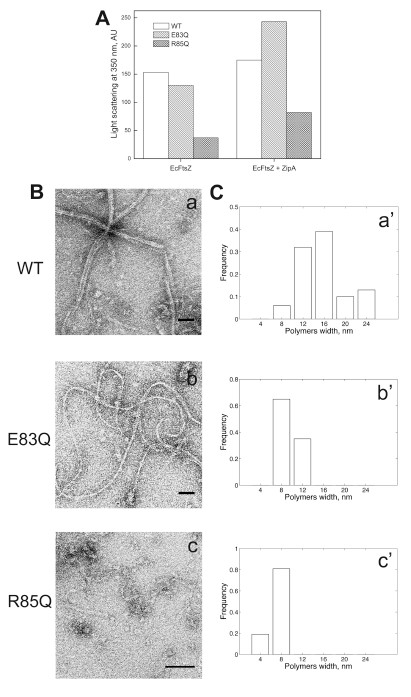
**Polymerization of E83Q and R85Q in presence of ZipA.** (**A**) The polymerization of the FtsZ mutants and wild type was followed by light scattering; the buffer and protein concentrations were the same as described in Figure 4. After a stable base line of the buffer containing 1mM GTP, with or without ZipA, FtsZ was added to initiate the polymerization reaction. The change in light scattering at 350 nm was recorded during the polymerization reaction and the difference between the base line and the maximum value reached after FtsZ addition is shown in the bar graph. (**B**) Electron microscopy of negatively stained polymers of wild type FtsZ, E83Q and R85Q polymerized as in (**A**). The samples for FtsZ WT, E83Q and R85Q were taken at 23 min, 53 min and 2h 50 min, respectively (see Additional file 1: Figure S4 for polymerization curves). Electron micrographs were taken at a magnification of 28,500 for wild type FtsZ and E83Q, and of 52,000 for R85Q. Scale bar, 50 nm. (**C**) Histogram of FtsZ polymers width distribution shows that the mutations E83Q and R85Q decrease the number of protofilaments per polymer. At least 31 polymers were measured for each case using ImageJ.

The polymerization reaction was started with the addition of the protein, and the reaction was followed for one hour for wild type and E83Q, while R85Q was followed for almost three hours due to the long lag period observed for the GTPase activity (Figure 4B). The mutant R85Q apparently did not form long filaments at the protein concentration used for FtsZ wild type and E83Q (Figure 5C), producing a small increment in turbidity that was stimulated around two-fold by ZipA Additional file 1: Figure S3a), whereas FtsZ wild type and E83Q showed a fast polymerization kinetics that could not be measured. E83Q formed polymers that remained in solution around three times longer than those formed by FtsZ wild type, with a rate of depolymerization 3.9 times slower. This increase in the time in which the filaments remain polymerized can be explained by the small apparent catalytic constant for the GTPase activity. The time traces of light scattering (Additional file 1: Figure S4) indicate that the polymerization and the subsequent depolymerization of FtsZ are altered by the mutations on the arginine 85 and glutamate 83. Additional file 1: Figure S5 shows that calcium increased the ligth scattering, and for FtsZ wild type and E83Q this was due to the formation of bundles, as shown in Additional file 1: Figure S6. On the other hand, R85Q presented a smaller increase in light scattering and no formation of bundles was observed.

Figure 5A shows the maximum light scattering value reached by FtsZ in 1 mM GTP in the presence and absence of ZipA. The mutants presented a decrease in the polymerization levels of ~20% and ~75% for E83Q and R85Q, respectively. In the presence of ZipA, the maximum light scattering value of FtsZ wild type increased slightly, (~20%, and bundles were observed by electron microscopy, Additional file 1: Figure S6), while for both mutants the increment was significantly larger: 90% and 150% for E83Q and R85Q, respectively (Figure 5A). In the absence of ZipA, FtsZ wild type polymerized into long and mostly straight filaments of 8 to 24 nm width (media around 14 nm) suggesting that the main population of these filaments consists of 4 assembled protofilaments of 4 nm width for each protofilament (Figure 5Ba and Ca'). E83Q polymerized into curved filaments with a width of 8 to 12 nm (Figure 5Bb and Cb'). The greatest effect was caused by the mutation R85Q, in which the polymers were mainly short and straight, and occasionally, spirals probably composed of single or double protofilaments were found on the grids (Figure 5Bc and Cc'). ZipA induced bundling of wt and E83Q, which is in agreement with previous studies [34]; however, we did not observe a clear bundling effect in the R85Q mutant, even given the increase in light scattering. The light scattering contribution of ZipA was subtracted, therefore we can rule out the possibility that the increase in the light scattering of R85Q is due to ZipA. Further characterization of R85Q will be done in a future study.

All the results together indicate that arginine 85 and glutamate 83 participate in the longitudinal and in the lateral interactions, shown by their reduced GTPase activity and the effect on polymerization, respectively. Interestingly, the addition of a stabilizer of the lateral interactions, such as ZipA to R85Q, did not induce bundles (Additional file 1: Figure S6) but stimulated the GTPase activity (Figure 4B and Additional file 1: Figure S3b), indicating that protofilaments are induced. On the other hand, in the case of E83Q, even though the protofilament lateral interactions were diminished (Figure 5B) and ZipA induced bundles that were thinner than those with FtsZ wild type, the GTPase activity was not greatly compromised. Therefore, we believe that the ability of FtsZ to form bundles, presumably through lateral interactions, is important to form the Z-ring in the cell, and consequently for cell division, a conclusion consistent with our immunofluorescence experiments (Figure 3) and cell viability results (Figure 2).

## Discussion

The aim of this work was to characterize the role of two conserved amino acid residues, E83 and R85, located in the bend of helix H3 at the lateral face of FtsZ (Figure 1A). Our results show that these amino acids are relevant for cell division, FtsZ polymerization, GTPase activity and stability of the polymers.

The properties of these FtsZ mutants were evaluated through their kinetics and critical concentrations for polymerization and GTPase activities in the absence and presence of ZipA and calcium. The FtsZ mutant proteins had the same secondary structure as the wild type form (within the experimental error) indicating an intact global fold. Interestingly, although the mutations are located far from the GTPase catalytic site (at the logitudinal interface within the filaments) both mutant proteins hydrolyzed GTP with less efficiency than wild type in the following order: WT > E83Q > R85Q. Compared to the wild type FtsZ, filaments with mutant proteins were thinner with altered curvatures, indicating that the mutations affect lateral interactions between FtsZ protofilaments. ZipA, which in wild type FtsZ is known to induce bundling without affecting GTPase activity, induced thinner bundles in E83Q, while in the case of R85Q was unable to cause bundling, suggesting that both glutamate 83 and arginine 85 are important for lateral interactions between protofilaments. R85Q filaments were short and abnormally bent, indicating that the mutation also affects the geometry of the longitudinal interactions. The formation of short filaments is consistent with the ability of R85Q to hydrolyze GTP, which requires longitudinal interaction between two FtsZ molecules [35-38]. Also the neighbor mutation D86K, which induces twined filaments, shows a reduction in GTPase activity further suggesting a role of this region in longitudinal interactions [11]. The long lag period of the progress curve of R85Q GTPase activity was suppressed by increasing the protein concentration, confirming that this activity was directly related to polymerization. This result could be explained by a reduction of the nucleation free energy over the free energy for the elongation process.

The bend in H3 is likely caused by the negatively-charged amino acids DED located just before the bend which destabilize the helix by negative charge repulsion. At the other end of the H3 bend, R85 is followed by D86; therefore, the positive charge of R85 could destabilize the positive dipole of the helix in this region. Thus, altering the charges in this region could induce a conformational change, explaining the effects of the R85Q, E83Q and D86K [24] mutations on the functionality of FtsZ.

Based on our results we hypothesize that, directly or via a subtle conformational change, the charges of amino acids located in the bend of the H3 helix are involved in longitudinal and lateral interactions. We infer that the negative charge of glutamate 83 weakens these interactions while the positive charge of arginine 85 stabilizes them, probably regulating the dynamics of the Z-ring. Thus, cells carrying FtsZ(E83Q) or FtsZ(R85Q) were affected in cell division and showed reduced survival and cell filamentation in agreement with previously described lateral-interaction-FtsZ mutants that could not complement the temperature sensitive *ftsZ84*[10]. We have quantified survival rates at the restrictive temperature (42°C) as a means to evaluate the effect of FtsZ mutations on cell division, similar to studies on the effects of proteins expression from unstable plasmids [39,40].

The E83Q and R85Q mutations diminished the apparent catalytic constant of GTPase activity by 60% and 95%, respectively. Presumably, the positive charge of R85 is involved in longitudinal and lateral interactions between protofilaments explaining the inhibitory effect of the R85Q mutation on GTPase activity and polymerization when compared to the E83Q or D86K mutants (Table 1) [11]. Both, longitudinal and lateral interactions are important in models for the conversion of GTP hydrolysis into mechanical energy during Z-ring constriction [8,25]. The observed contraction of tubular liposomes in the presence of GTP and a chimeric, membrane-attached FtsZ indicates that polymerization of FtsZ is sufficient to generate a mechanical force to bend the membrane. Our results indicate that H3 bend mutations affecting longitudinal and lateral interactions *in vitro* reduce cell viability *in vivo* while they cause cell filamentation. Cell filamentation and the immunofluorescence analysis (Figure 3) together with the polymerization results support a model of a Z-ring composed of several polymers that are recruited to the septation site by lateral interactions, as has been found *in vivo*[13].

**Table 1:** Summary of FtsZ mutations and their effects on cell viability, GTPase activity and polymer morphology

**Location of the mutation**		**Viability**^***b***^**, %**	**GTPase activity**^***c***^**, %**	**Polymer morphology**^***d***^	**Ref.**
**Lateral side**^***a***^	**aa, nº**			**ZipA**	
	WT	100	100	B	This study
Left	E83Q	10	40	B	This study
Left	R85Q	0.5	5	S	This study
Left	D86K	N	49	Nd	[11]
Right	E250A	N	67	Nd	[11]

^*a*^The mutations are positioned on the surface and their location on the structure are shown in Figure 1. Left: the left lateral surface; Right: the right lateral surface.

aa, nº = amino acid number.

^*b*^The % viability was determined from data of Figure 2. N = no growth of *ftsZ84* at the restrictive temperature.

^*c*^GTPase activity was measured with 12 μM of protein in all cases.

^*d*^B, bundles; S, short filaments; Nd, not determined. The shape of the polymers was determined by electron microscopy (Figure 5).

## Conclusion

This study describes the role of the positively charged arginine 85 and the negatively charged glutamate 83 in longitudinal and lateral interactions to form FtsZ protofilaments and two-dimensional polymers of protofilaments, respectively. We also show that GTPase activity could be fine-tuned by the net charge of the H3 helix bend. Finally, our results suggest that the conserved amino acids E83Q and R85Q located in the bend of H3 are involved in the formation of a functional Z-ring.

## Methods

### Bacterial strains and plasmids

Strain C41(DE3), cured from C41/pHis17-MJ0370 [41], was used to express *E. coli* FtsZ WT and mutants. VIP2(DE3)/pLAR9 was used to evaluate the functionality of FtsZ *in vivo*. VIP2(DE3)/pLAR9 is VIP2/pLAR9 [31] lysogenized with the λDE3 Lysogenization Kit (Novagen).

pMFV57 was constructed from pMFV56 [31] by cloning the EcoRV *β-lactamase* fragment into the kanamycin^r^ (kan) gene. The *β-lactamase* gene was generated by PCR using the following primers: 5′-CATTCAAATATGGATCCGCTCATG-3′ and 5′-ACCAATGCTTAATCAGTGAGG-3′. FtsZ E83Q and R85Q mutants were constructed using the QuikChange XL Site-Directed Mutagenesis Kit (Stratagene) and the pairs of primers used to generate the mutations were the following: 5′-GCAATGCGGCTGATCAGGATCGCGATGCATTGC-3′ and 5′-GCAATGCATCGCGATCCTGATCAGCCGCATTGC-3′; 5′-GCGGCTGATGAGGATCAGGATGCATTGCGTGCG-3′ and 5′-CGCACGCAATGCATCCTGATCCTCATCAGCCGC-3′, respectively. The presence of the *ftsZ* gene mutations was confirmed by DNA sequencing and the new plasmids were named pMFV-E83Q and pMFV-R85Q. These constructions are derivatives of the pET28 expression system; FtsZ expression is thus inducible by the addition of IPTG.

### Protein purification

*E. coli* FtsZ(WT), FtsZ(E83Q) and FtsZ(R85Q) mutants were purified as described previously [42] with some modifications. *E. coli* C41(DE3) carrying the appropriate pMFV-plasmid derivative was grown in LB-ampicilin (amp) with shaking at 37°C. When the OD_550_ reached 0.5, IPTG was added to a final concentration of 0.5 mM and the culture was grown for a further 3 h. Harvested cells were washed and then suspended in buffer A (50 mM Tris–HCl, pH 7.9, 50 mM KCl, 1 mM ethylenediaminetetraacetic acid (EDTA), 10% glycerol). After lysis by sonication (Sonifier Cell Disruptor Model W185, 1 cm diameter), the suspension was centrifuged at 28,000 rpm, at 4°C for 90 min (Beckman L5-75B Ultracentrifuge, rotor type 30) to remove the membrane fraction and the supernatant was collected. The proteins were precipitated by adding 35% ammonium sulfate, centrifuged in the same rotor at 15,000 rpm for 20 min and the pellet was dissolved in buffer A. The dialyzed ammonium sulfate fraction was loaded onto a Q-Sepharose column (25 cm × 4.6 mm) and eluted with a 50 mM – 1 M gradient of KCl at a flow rate of 1 ml/min. The FtsZ fractions were loaded onto a Sephacryl S-400 column and eluted with buffer A at a flow rate of 1 ml/min. The peak fractions were pooled, concentrated to 10 – 15 mg protein/ml using Centriprep, fast frozen and stored at −80°C. The *E. coli* FtsZ obtained was pure, as determined by SDS-PAGE gels and western blotting.

### *In vivo* cell survival assay

To evaluate the functionality of the FtsZ(E83Q) and FtsZ(R85Q), the viability of cells expressing these mutants was determined using the strain *E. coli* VIP2(DE3)/pLAR9 (also called VIP5). In this strain the *ftsZ* chromosomal copy is interrupted by an insertion (resistant to kanamycin (kan)), and complemented with a copy encoded on the thermosensitive plasmid pLAR9, resistant to chloramphenicol (cam) [31]. The assay was performed as described by Díaz-Espinoza et al. [39]. Briefly, overnight cultures of VIP5 transformed with or without pMFV-WT, pMFV-E83Q or pMFV-R85Q, were diluted 1:100 in LB media containing 100 μg/ml amp and grown at 30°C until the OD_550_ reached 0.5 – 0.6. Serial dilutions of this culture plated on LB-amp were incubated, at both the permissive (30°C) and non-permissive temperatures (42°C). The next day, the survival was determined for wtFtsZ and each of the mutants.

### GTPase activity

GTPase activity was monitored by determining the area under the peaks corresponding to GDP and GTP from the HPLC chromatograms obtained after loading the de-proteinized reaction samples onto a SUPERCOSIL LC-18-DB column (SUPELCO) as previously described [43]. FtsZ was incubated for 2 min in polymerization buffer (50 mM Mes pH 6.5, 50 mM KCl, 10 mM MgCl_2_) at 30°C and the reaction was started by addition of GTP (1 mM final concentration). At different times, 100 μl samples were withdrawn and transferred to 8 μl 70% perchloric acid and neutralized by the addition of 100 μl 1.2 M K_2_CO_3_. After degasification in a LABCONCO Speed-Vac at room temperature for 5 min, the reaction was centrifuged at 14,000 rpm for 15 min and a 20 μl aliquot of the supernatant was loaded onto the SUPERCOSIL LC-18-DB column and eluted at 1 ml/min using a buffer containing 0.2 M K_2_HPO_4_, 0.1 M acetic acid and 4 mM tetrabutylammonium phosphate monobasic (TBAP) in an HPLC. GDP and GTP were detected at 260 nm by a UV detector system. Alternatively, the inorganic phosphate product of GTP hydrolysis was determined using the colorimetric method of malachite green [44]. In brief, the polymerization sample (70 μL) was mixed with perchloric acid (10% final concentration). After 5 minutes, 50 μL of this solution were mixed in a vortex for 1 min with 800 μl malachite green reagent 0.045%. 100 μl of citrate (34%) were added and the solution was kept on ice for 20 min before the measurement of the absorbance at 630 nm.

### *E. coli* FtsZ polymerization by light scattering

Assembly of FtsZ was monitored using the Luminescence Spectrometer LS 50 (Perkin Elmer) as described previously [33]. Excitation and emission wavelengths were set to 350 nm, with a slit width of 5 nm. 12.5 μM FtsZ was incubated in polymerization buffer at 30°C in a fluorescence cuvette with a 1 cm path length and after 2 min of data collection, GTP was added to a final concentration of 1 mM.

### Immunofluorescence of *E. coli* FtsZ and ZipA

To label FtsZ and ZipA, cells were treated following the method described by Rueda et al. [45]. This method has three steps: fixation, permeabilization and development with specific antibodies. A) Fixation. In order to compare the localization of the proteins at 30°C and 42°C, duplicated cellular cultures of VIP2/pLAR9, complemented with FtsZ(WT) and the mutants FtsZ(E83Q) or FtsZ(R85Q) were grown. The cultures grown at 30°C contained the antibiotics kan, amp and cam, while cultures grown at 42°C only contained kan and amp. The negative control was the strain VIP2/pLAR9 without pMFV57, and no amp was added to the culture. After the cell culture reached an OD_600_ of 0.5, one of the duplicated tubes was incubated at 42°C and the growth continued for another 4 h. The cells were fixed with 0.75% formaldehyde and incubated for 10 min at room temperature followed by 30 min on ice. The fixed cells were washed with PBS and suspended in buffer GTE (20 mM Tris–HCl pH 7.5, 50 mM glucose and 10 mM EDTA). B) Permeabilization. 8 μg/ml final concentration of lysozyme were added to 30 μl of fixed cells, placed on a glass slide coated with poly-L-lysine and incubated at room temperature for 2–3 min and washed with PBS. C) Development was carried out with specific antibodies against FtsZ and ZipA. The unspecific binding sites were blocked by adding 3% BSA in PBS for 15 min at room temperature and this solution was replaced by a solution containing an appropriate dilution of the primary polyclonal antibody (anti-FtsZ or anti-ZipA). After an overnight incubation at 4°C, the cells were washed with PBS and incubated for 2 h with the secondary antibody (anti-rabbit labeled with Alexa 488, Invitrogen) at room temperature in darkness. After several washes with PBS, a drop of antifading (Vectors Lab) was added and the samples covered with a cover slide. Cells were observed in a confocal microscope (Leica TCS SP2).

### Electron microscopy

Staining and visualization of the FtsZ polymers were carried out in a JEOL JEM-100SX electron microscope. The FtsZ polymers in absence or presence of ZipA were incubated at 30°C in polymerization buffer with 1 mM GTP. After the maximal polymerization was reached (as detected by light scattering), 10 μl of the polymerization reaction was placed on a carbon-coated grid for 1 min and stained with 1% of aqueous uranyl acetate. Electron micrographs were taken at different magnifications. Each picture was digitalized directly from the film and the width of the polymers was measured using the computer program ImageJ 1.34 s ( http://rsb.info.nhi.gov/ij/download.htlm).

## Endnotes

^a^ The 3D structure of the *E. coli* FtsZ model was built with the program MODELLER v6.2 using as template the crystal structures of *M. jannaschii* FtsZ (1FSZ.pdb) and *P. aureginosa* FtsZ (1OFU.pdb). The program gave a rms value of 0.31 Å for the structural fit. The good quality of the model was assessed with the computer programs PROSA II and Verify 3D (web site http://nhiserver.mbi.ucla.edu/Verify_3D/).

## Competing interest

The authors declare no conflict of interest.

## Authors’ contributions

JYS contributed to the conception and design of the study, and conducted the experiments. OM and RL conceived the study, designed the experiments, analyzed and interpreted the data. WV designed, conducted and supervised the electron and confocal microscopy experiments. JYS, WV, RL and OM wrote the manuscript. All authors have no conflicts of interest to report. All authors read and approved the final manuscript.

## Supplementary Material

Additional file 1: Figure S1 Cellular localization and distribution of FtsZ WT and the mutants E83Q and R85Q. **Figure S2.** Localization and distribution of ZipA in cells expressing FtsZ WT and mutants E83Q and R85Q. **Figure S3.** Effect of ZipA on the polymerization of FtsZ R85Q using light scattering and GTPase activity. **Figure S4.** Polymerization kinetics of FtsZ WT and the mutants E83Q and R85Q detected by light scattering. **Figure S5.** Effect of Ca^2+^ on the polymerization of FtsZ WT and the mutants E83Q and R85Q using light scattering. **Figure S6.** Effect of Ca^2+^ and ZipA on the polymers morphology of FtsZ WT and the mutants E83Q and R85Q. Click here for file

## References

[B1] Geissler B Elraheb D Margolin W A gain-of-function mutation in ftsA bypasses the requirement for the essential cell division gene zipA in Escherichia coli Proc Natl Acad Sci USA 2003 100 4197 4202 10.1073/pnas.0635003100 12634424 PMC153070

[B2] Liu Z Mukherjee A Lutkenhaus J Recruitment of ZipA to the division site by interaction with FtsZ Mol Microbiol 1999 31 1853 1861 10.1046/j.1365-2958.1999.01322.x 10209756

[B3] Vicente M Rico AI The order of the ring: assembly of Escherichia coli cell division components Mol Microbiol 2006 61 5 8 10.1111/j.1365-2958.2006.05233.x 16824090

[B4] Addinall SG Bi E Lutkenhaus J FtsZ ring formation in fts mutants J Bacteriol 1996 178 3877 3884 8682793 10.1128/jb.178.13.3877-3884.1996 PMC232649

[B5] Sun Q Yu XC Margolin W Assembly of the FtsZ ring at the central division site in the absence of the chromosome Mol Microbiol 1998 29 491 503 10.1046/j.1365-2958.1998.00942.x 9720867

[B6] Osawa M Anderson DE Erickson HP Reconstitution of contractile FtsZ rings in liposomes Science 2008 320 792 794 10.1126/science.1154520 18420899 PMC2645864

[B7] Allard JF Cytrynbaum EN Force generation by a dynamic Z-ring in Escherichia coli cell division Proc Natl Acad Sci USA 2009 106 145 150 10.1073/pnas.0808657106 19114664 PMC2629190

[B8] Lan G Daniels BR Dobrowsky TM Wirtz D Sun SX Condensation of FtsZ filaments can drive bacterial cell division Proc Natl Acad Sci USA 2009 106 121 126 10.1073/pnas.0807963106 19116281 PMC2629247

[B9] Erickson HP Modeling the physics of FtsZ assembly and force generation Proc Natl Acad Sci USA 2009 106 9238 9243 10.1073/pnas.0902258106 19478069 PMC2695047

[B10] Koppelman M-C Arsman MEG Postmus J Pas E Muijers AO Scheffers D-J Nanninga N den Blaauwen T R-174 of Escherichia coli FtsZ is involved in membrane interaction and protofilament bundling, and is essential for cell division Mol Microbiol 2004 51 645 657 14731269 10.1046/j.1365-2958.2003.03876.x

[B11] Lu C Stricker J Erickson HP Site-specific mutations of FtsZ–effects on GTPase and in vitro assembly BMC Microbiol 2001 1 7 10.1186/1471-2180-1-7 11394965 PMC32248

[B12] Monahan LG Robinson A Harry JE Lateral FtsZ association and the assembly of the cytokinetic Z ring in bacteria Mol Microbiol 2009 74 1004 1007 10.1111/j.1365-2958.2009.06914.x 19843223

[B13] Fu G Huang T Buss J Coltharp C Hensel Z Xiao J In vivo structure of the E. coli FtsZ-ring revealed by photoactivated localization microscopy (PALM) PLoS One 2010 5 e12682 10.1371/journal.pone.0012682 20856929 PMC2938336

[B14] Erickson HP Taylor DW Taylor KA Bramhill D Bacterial cell division protein FtsZ assembles into protofilament sheets and minirings, structural homologs of tubulin polymers Proc Natl Acad Sci USA 1996 93 519 523 10.1073/pnas.93.1.519 8552673 PMC40269

[B15] Hale CA Rhee AC de Boer PA ZipA-induced bundling of FtsZ polymers mediated by an interaction between C-terminal domains J Bacteriol 2000 182 5153 5166 10.1128/JB.182.18.5153-5166.2000 10960100 PMC94664

[B16] Bramhill D Thompson CM GTP-dependent polymerization of Escherichia coli FtsZ protein to form tubules Proc Natl Acad Sci USA 1994 91 5813 5817 10.1073/pnas.91.13.5813 8016071 PMC44087

[B17] Lowe J Amos LA Helical tubes of FtsZ from Methanococcus jannaschii Biol Chem 2000 381 993 999 11076032 10.1515/BC.2000.122

[B18] Lu C Reedy M Erickson HP Straight and curved conformations of FtsZ are regulated by GTP hydrolysis J Bacteriol 2000 182 164 170 10.1128/JB.182.1.164-170.2000 10613876 PMC94253

[B19] Gündogdu ME Kawai Y Pavlendova N Ogasawara N Errington J Scheffers D-J Hamoen LW Large ring polymers align FtsZ polymers for normal septum formation EMBO J 2011 30 617 626 10.1038/emboj.2010.345 21224850 PMC3034016

[B20] Singh JK Makde RD Kumar V Panda D SepF Increases the Assembly and bundling of FtsZ polymers and stabilizes FtsZ protofilaments by binding along its length J Biol Chem 2008 283 31116 31124 10.1074/jbc.M805910200 18782755 PMC2662178

[B21] Erickson HP Stoffler D Protofilaments and rings, two conformations of the tubulin family conserved from bacterial FtsZ to alpha/beta and gamma tubulin J Cell Biol 1996 135 5 8 10.1083/jcb.135.1.5 8858158 PMC2121013

[B22] Li Z Trimble MJ Brum YU Jensen GJ The structure of FtsZ filaments in vivo suggest a force-generation role in cell division EMBO J 2007 26 4694 4708 10.1038/sj.emboj.7601895 17948052 PMC2080809

[B23] Huecas S Llorca O Boskovic J Martin-Benito J Valpuesta JM Andreu JM Energetics and geometry of FtsZ polymers: nucleated self-assembly of single protofilaments Biophys J 2008 94 1796 1806 10.1529/biophysj.107.115493 18024502 PMC2242775

[B24] Oliva MA Huecas S Palacios JM Martin-Benito J Valpuesta JM Andreu JM Assembly of archaeal cell division protein FtsZ and a GTPase-inactive mutant into double-stranded filaments J Biol Chem 2003 278 33562 33570 10.1074/jbc.M303798200 12807911

[B25] Baumann P Jackson SP An archaebacterial homologue of the essential eubacterial cell division protein FtsZ Proc Natl Acad Sci USA 1996 93 6726 6730 10.1073/pnas.93.13.6726 8692886 PMC39094

[B26] de Pereda JM Leynadier D Evangelio JA Chacon P Andreu JM Tubulin secondary structure analysis, limited proteolysis sites, and homology to FtsZ Biochemistry 1996 35 14203 14215 10.1021/bi961357b 8916905

[B27] Nogales E Whittaker M Milligan RA Dowing KH High-resolution model of the microtubule Cell 1999 99 79 88 9989499 10.1016/s0092-8674(00)80961-7

[B28] Schlieper D Oliva MA Andreu JM Lowe J Structure of bacterial BtubA/B: Evidence or horizontal gene transfer Proc Natl Acad Sci 2005 102 9170 9175 10.1073/pnas.0502859102 15967998 PMC1166614

[B29] Nogales E Downing KH Amos LA Lowe J Tubulin and FtsZ form a distinct family of GTPases Nat Struct Biol 1998 5 451 458 10.1038/nsb0698-451 9628483

[B30] Pilhofer M Ladinsky MS McDowall W Petroni G Jensen GJ Microtubules in bacteria: ancient tubulins build a five protofilament homolog of the eukaryotic cytoskeleton PLoS Biol 2011 9 e1001213 10.1371/journal.pbio.1001213 22162949 PMC3232192

[B31] Pla J Sanchez M Palacios P Vicente M Aldea M Preferential cytoplasmic location of FtsZ, a protein essential for Escherichia coli septation Mol Microbiol 1991 5 1681 1686 10.1111/j.1365-2958.1991.tb01915.x 1943703

[B32] RayChaudhuri D ZipA is a MAP-Tau homolog and is essential for structural integrity of the cytokinetic FtsZ ring during bacterial cell division EMBO J 1999 18 2372 2383 10.1093/emboj/18.9.2372 10228152 PMC1171320

[B33] Mukherjee A Lutkenhaus J Analysis of FtsZ assembly by light scattering and determination of the role of divalent metal cations J Bacteriol 1999 181 823 832 9922245 10.1128/jb.181.3.823-832.1999 PMC93448

[B34] Lowe J Amos LA Tubulin-like protofilaments in Ca2+−induced FtsZ sheets EMBO J 1999 18 2364 2371 10.1093/emboj/18.9.2364 10228151 PMC1171319

[B35] Mukherjee A Lutkenhaus J Dynamic assembly of FtsZ regulated by GTP hydrolysis EMBO J 1998 17 462 469 10.1093/emboj/17.2.462 9430638 PMC1170397

[B36] Mukherjee A Lutkenhaus J Guanine nucleotide-dependent assembly of FtsZ into filaments J Bacteriol 1994 176 2754 2758 8169229 10.1128/jb.176.9.2754-2758.1994 PMC205420

[B37] Mukherjee A Saez C Lutkenhaus J Assembly of an FtsZ mutant deficient in GTPase activity has implications for FtsZ assembly and the role of the Z ring in cell division J Bacteriol 2001 183 7190 7197 10.1128/JB.183.24.7190-7197.2001 11717278 PMC95568

[B38] Scheffers DJ de Wit JG den Blaauwen T Driessen AJ GTP hydrolysis of cell division protein FtsZ: evidence that the active site is formed by the association of monomers Biochemistry 2002 41 521 529 10.1021/bi011370i 11781090

[B39] Diaz-Espinoza R Garcés AP Arbildúa JJ Montecinos F Brunet JE Lagos R Monasterio O Domain folding and flexibility of Escherichia coli FtsZ determined by tryptophan site-directed mutagenesis Protein Sci 2007 16 1543 1556 10.1110/ps.072807607 17656575 PMC2203363

[B40] Wang X Huang J Mukherjee A Cao C Lutkenhaus J Analysis of the interaction of FtsZ with itself, GTP and FtsA J Bacteriol 1997 179 5551 5559 9287012 10.1128/jb.179.17.5551-5559.1997 PMC179428

[B41] Lowe J Amos LA Crystal structure of the bacterial cell-division protein FtsZ Nature 1998 391 203 206 10.1038/34472 9428770

[B42] Mukherjee A Lutkenhaus J Purification, assembly, and localization of FtsZ Methods Enzymol 1998 298 296 305 9751889 10.1016/s0076-6879(98)98026-0

[B43] Nova E Montecinos F Brunet JE Lagos R Monasterio O 4',6-Diamidino-2-phenylindole (DAPI) induces bundling of Escherichia coli FtsZ polymers inhibiting the GTPase activity Arch Biochem Biophys 2007 465 315 319 10.1016/j.abb.2007.06.032 17678870

[B44] Lanzetta PA Alvarez LJ Reinach PS Candia OA An improved assay for nanomole amounts of inorganic phosphate Anal Biochem 1979 100 95 97 10.1016/0003-2697(79)90115-5 161695

[B45] Rueda S Vicente M Mingorance J Concentration and assembly of the division ring proteins FtsZ, FtsA, and ZipA during the Escherichia coli cell cycle J Bacteriol 2003 185 3344 3351 10.1128/JB.185.11.3344-3351.2003 12754232 PMC155373

