# Supplementary material for: Glutamate 83 and arginine 85 of helix H3 bend are key residues for FtsZ polymerization, GTPase activity and cellular viability of Escherichia coli: lateral mutations affect FtsZ polymerization and E. coli viability
Source: BMC Microbiol. 2013 Feb 5;13:26. doi: 10.1186/1471-2180-13-26 (PMC3626584; doi:10.1186/1471-2180-13-26)
Supplement: Additional file 1: Figure S1 — Cellular localization and distribution of FtsZ WT and the mutants E83Q and R85Q. Figure S2. Localization and distribution of ZipA in cells expressing FtsZ WT and mutants E83Q and R85Q. Figure S3. Effect of ZipA on the polymerization of FtsZ R85Q using light scattering and GTPase activity. Figure S4. Polymerization kinetics of FtsZ WT and the mutants E83Q and R85Q detected by light scattering. Figure S5. Effect of Ca2+ on the polymerization of FtsZ WT and the mutants E83Q and R85Q using light scattering. Figure S6. Effect of Ca2+ and ZipA on the polymers morphology of FtsZ WT and the mutants E83Q and R85Q. [file 1471-2180-13-26-S1.pdf]

ADDITIONAL FILE

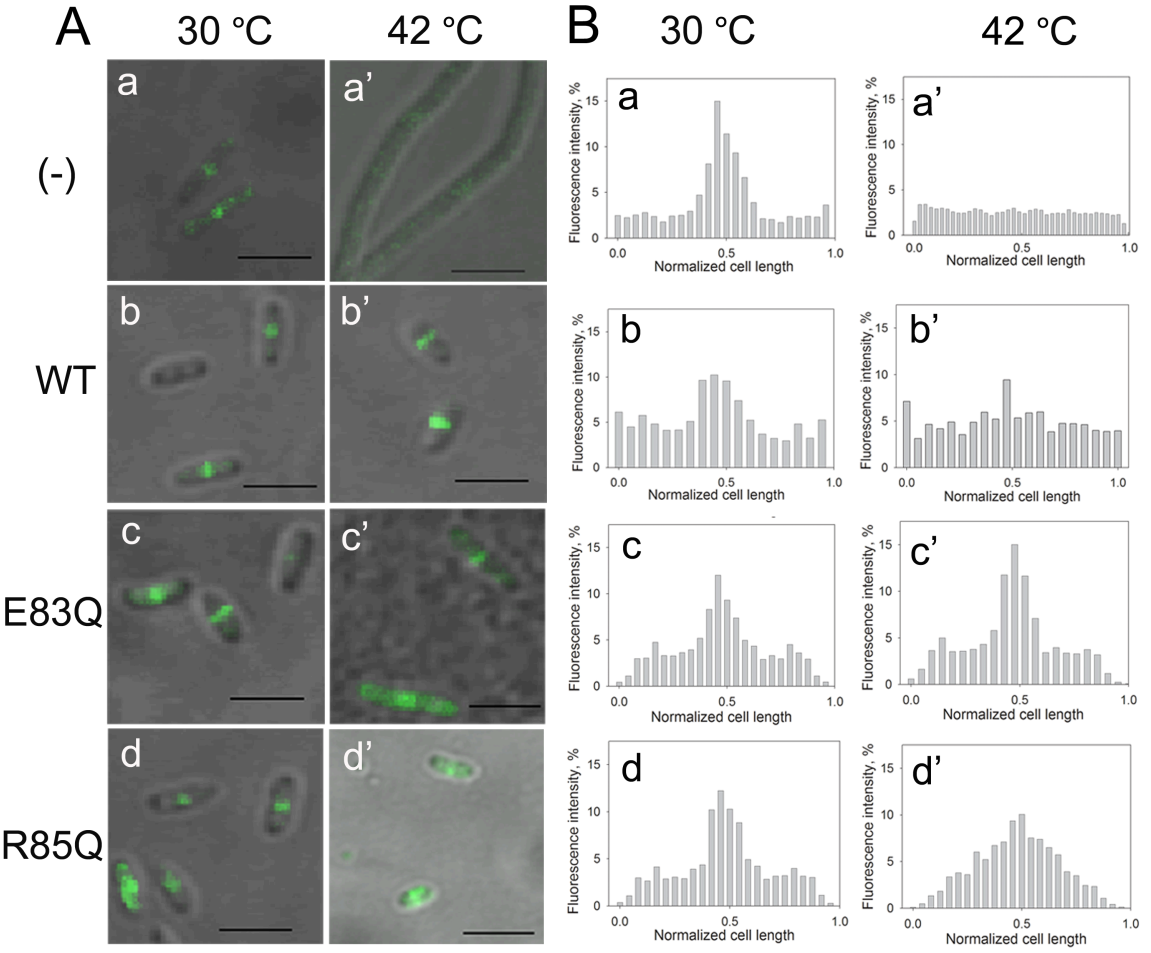

AF Figure 1. Cellular localization and distribution of FtsZ(E83Q) and FtsZ(R85Q). (**A)** lmmunofluorescence detection of FtsZ in VIP5 (-) cells (a [67] and a' [52]), expressing wild-type FtsZ (b [18] and b' [45]), FtsZ(E83Q) (c [75] and c' [37]) or FtsZ(R85Q) (d [57] and d' [44]). Number of cells analyzed is within the square brackets. Immunofluorescence images are overlaid with a bright field image. FtsZ, green. Bar, 5 μm. (**B**) Histogram of FtsZ distribution along the longitudinal axis of the VIP5 (-) cells (a and a') expressing wild-type FtsZ (b and b') and the mutants FtsZ(E83Q) (c and c') or FtsZ(R85Q) (d and d'). The fluorescence intensity profiles between the cell poles were generated with ImageJ (http://rsb.info.nih.gov/ij/download.html). The length of cells was normalized between 0 and 1 using a script developed in our laboratory. The expression of FtsZ mutants at early stationary phase diminished cell filamentation.

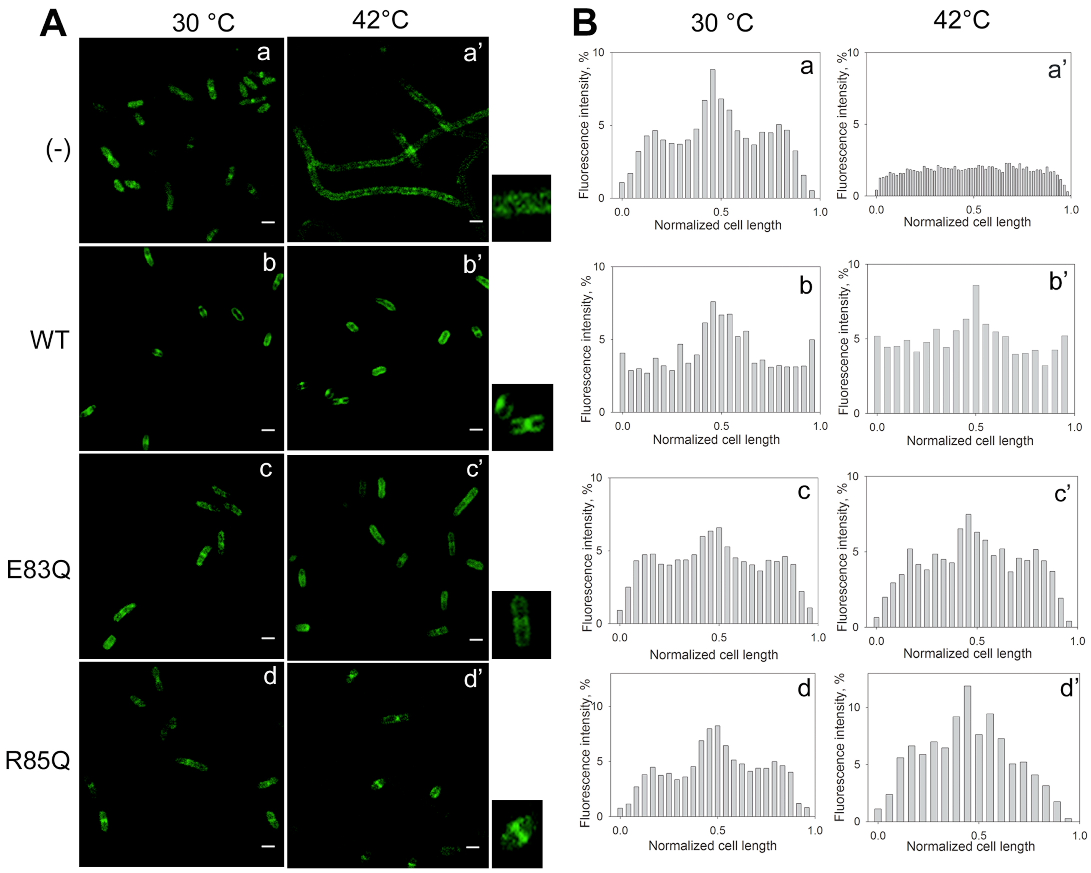

AF Figure 2. Localization and distribution of ZipA in cells expressing FtsZ(E83Q) or FtsZ(R85Q). (**A**) Immunofluorescence of ZipA in VIP5 (-) cells (a and a' [51]) expressing wild-type FtsZ (b [45] and b' [44]), FtsZ(E83Q) (c [65] and c' [54]) or FtsZ(R85Q) (d [46] and d' [45]). Number of cells analyzed is within the square brackets. ZipA, green. Bar, 5 μm. The right sites show zoomed images of representative cells. (**B**) Histogram of ZipA distribution along the longitudinal axis of VIP5 cells (-) (a and a') expressing wild-type FtsZ (b and b'), FtsZ(E83Q) (c and c') or FtsZ(R85Q) (d and d'). The fluorescence intensity profiles were generated as described in AF Fig. 1. The distribution of ZipA follows that of FtsZ mutants indicating that both mutations in FtsZ do not affect its interaction with ZipA.

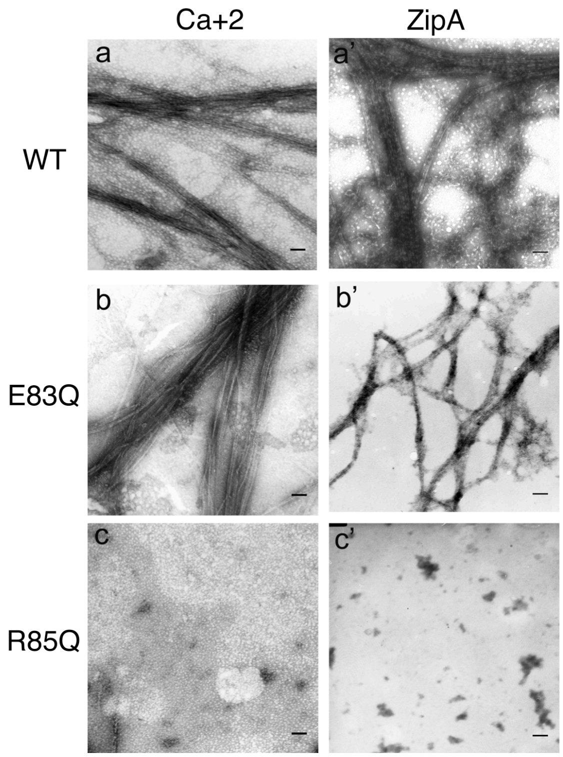

AF. Figure 3. Effect of calcium (Ca^{+2}) and ZipA protein on the polymers morphology of FtsZ wild type (WT) and mutants E83Q and R85Q. Electron microscopy (EM) shows that calcium and ZipA induce the formation of the bundle for the FtsZ WT and the mutant E83Q, which presumably is mediated by lateral interactions. Whereas calcium nor ZipA had bundling effect for the mutant R85Q. Sample for EM was prepared similar to Fig 6 from the main text. 5μM of EcFtsZ WT (a and a') and mutants E83Q (b and b') and R85Q (c and c') polymerized in presence of 1mM GTP, in presence of 10 mM Ca^{+2} (a, b and c) or 5 μM ZipA (a', b' and c'). Scale bar, 100 nm.

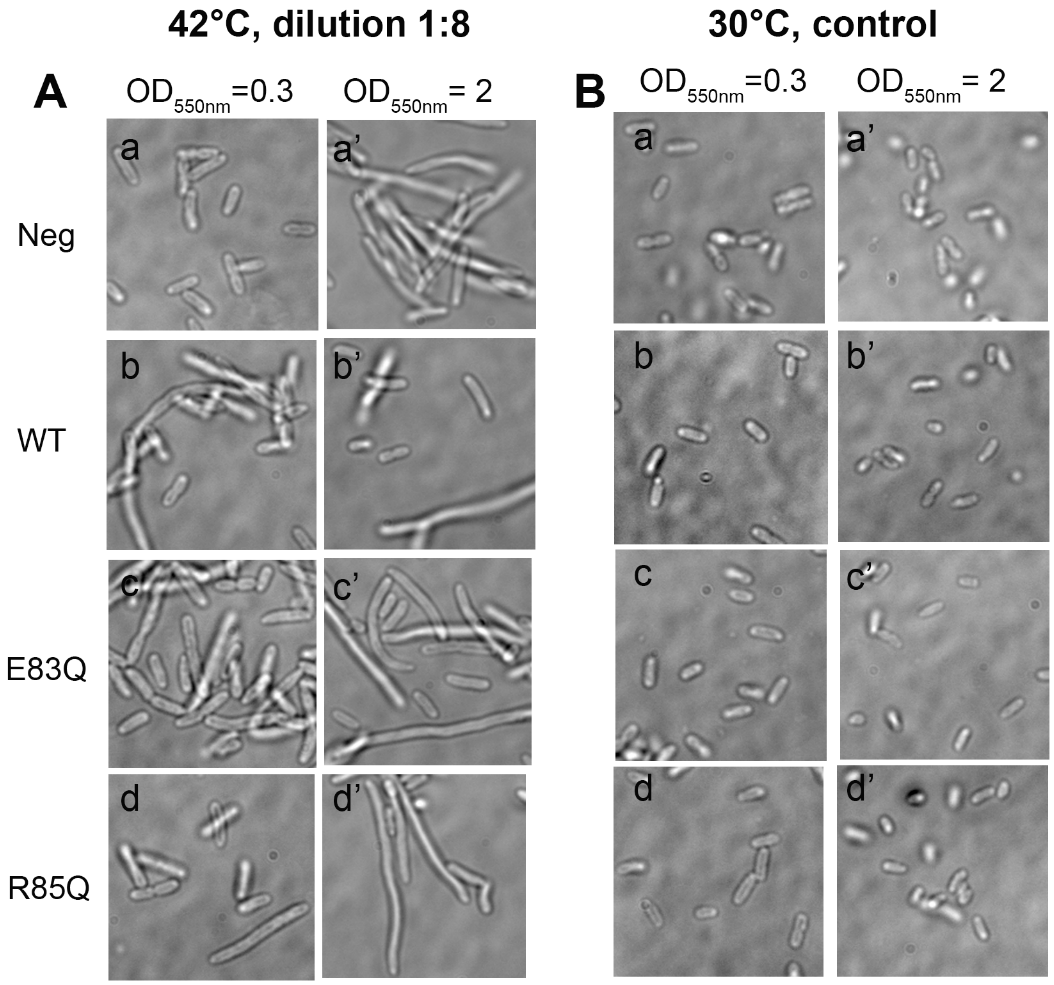

AF Figure 4. Morphology of cells expressing FtsZ wild type (WT) and mutants E83Q and R85Q with (30°C) and without (42°C) co-expression of FtsZ wild type. A) Cell cultures were grown at 30°C until OD_{550nm} reached ~0.5, diluted 1:8 in LB and transferred to 42°C for further growth. Cell samples were taken at the OD_{550nm} ~0.3 and ~2, fixed and imaged. Bright field image shows that cells form filaments at both exponential (OD_{550nm} ~0.3) and stationary (OD_{550nm} ~2) growth phase (Ab-d and Ac'-d'). In contrast to the morphology of cells with 1:4 dilution (Fig. 3A), the filamentous cells don't revert to the "normal" morphology at the stationary phase. B) As a control, cell grown at 30°C did not form filaments (a-d, a'-d'). Cell samples were taken at OD_{550nm} ~0.3 and ~2, fixed and imaged. Scale Bar, 5μm.

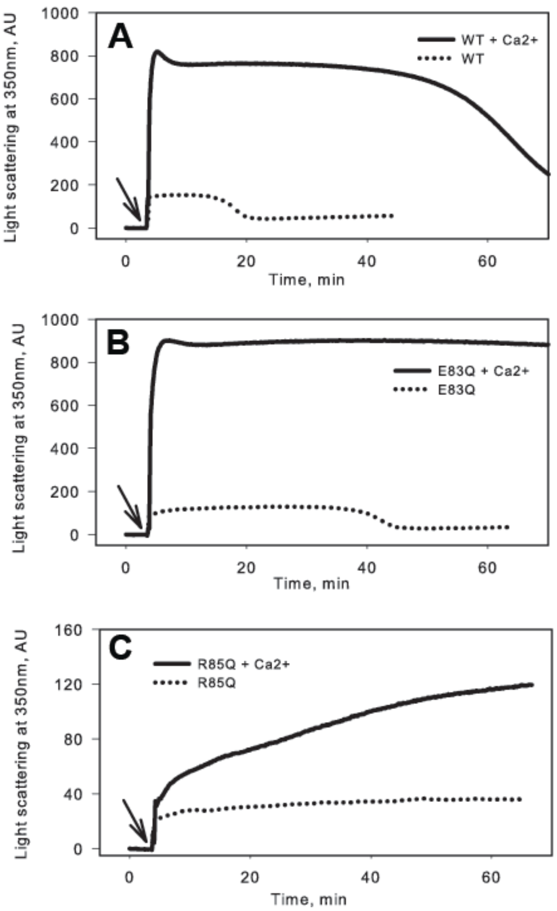

AF Figure 5. Effect of Ca^{2+} on the polymerization of FtsZ wild type (WT) and the mutants E83Q and R85Q using light scattering. Ca2+ induced the polymerization by 5 and 7 times of EcFtsZ WT (A) and mutant FtsZ E83Q (B), respectively (solid line). In contrast to the WT and the mutant E83Q, Ca^{2+} increased the light scattering of the mutant R85Q (C) much slower and less, and we could not attribute this increment to the polymer formation judged by the EM experiments (AF Fig. 1c). The polymerization reaction mixture was that indicated in Method and it was followed at 30°C by light scattering at 350 nm. The reaction was started with the addition of 12 μM protein (arrow) to the solution with (solid line) and without (dotted line) Ca^{2+} in the polymerization buffer.

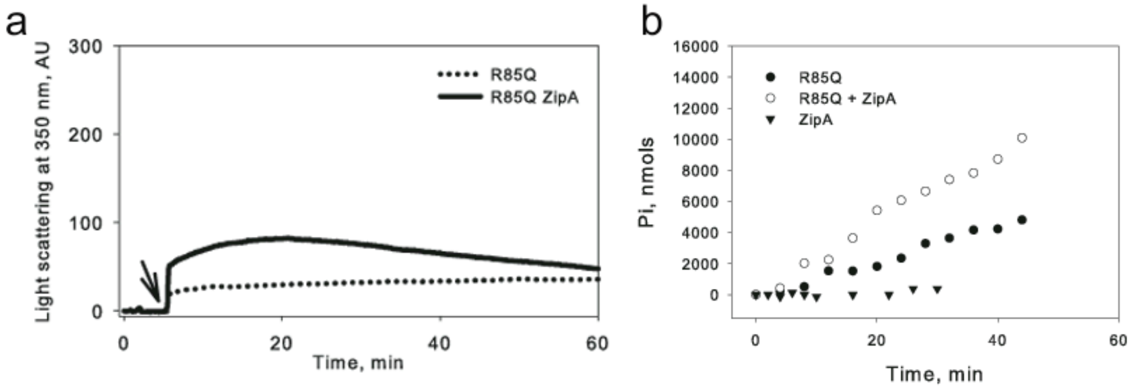

AF Figure 6. Effect of ZipA on the polymerization of FtsZ R85Q using light scattering and GTPase activity. ZipA induced the polymerization of FtsZ R85Q approximately 2 times (a, solid line) compared to its absence (a, dashed line). In contrast to the WT and the mutant E83Q (Fig 5C), ZipA enhanced the GTPase activity of the mutant R85Q (b). The arrow indicates the addition of FtsZ(R85Q).

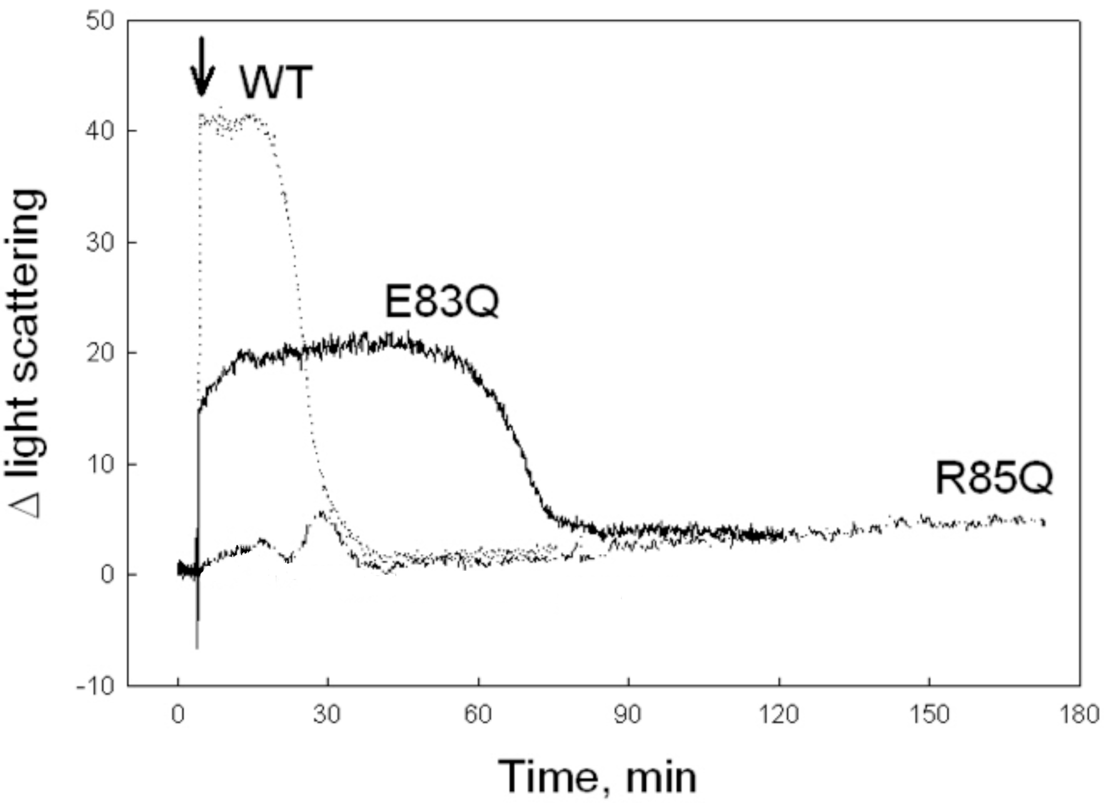

AF Figure 7. Polymerization kinetics of FtsZ detected by light scattering. FtsZ WT and the mutants FtsZ E83Q and FtsZ R85Q were polymerized in the same experimental conditions used to measure the GTPase activity, as shown in figure 4A. Polymerization was followed by 90° light scattering at 350 nm. The arrow indicates the addition of 1 mM GTP. The plateau of the R85Q mutant was reached after 150 min. The plateau of E83Q was longer than that of WT. Under these experimental conditions the light scattering value at the plateau region of E83Q was around 50% respect to that of the WT, whereas R85Q only reached around 12%.
